# PFGE and AFLP genotyping of *Staphylococcus aureus* subsp*. anaerobius* isolated from goats with Morel’s disease

**DOI:** 10.1007/s00203-012-0844-8

**Published:** 2012-09-14

**Authors:** O. Szaluś-Jordanow, D. Chrobak, M. Pyrgiel, A. Lutyńska, J. Kaba, M. Czopowicz, L. Witkowski, M. Kizerwetter-Świda, M. Binek, T. Frymus

**Affiliations:** 1 Department of Small Animal Disease with Clinic, Faculty of Veterinary Medicine, Warsaw University of Life Sciences, Nowoursynowska 159c, 02-776 Warsaw, Poland; 2 Department of Preclinical Sciences, Faculty of Veterinary Medicine, Warsaw University of Life Sciences, Ciszewskiego 8, 02-786 Warszawa, Poland; 3 Department of Sera and Vaccine Evaluation, National Institute of Hygiene, Chocimska 24, 01-172 Warsaw, Poland; 4 Department of Large Animal Disease with Clinic, Faculty of Veterinary Medicine, Warsaw University of Life Sciences, Nowoursynowska 159c, 02-776 Warsaw, Poland

**Keywords:** PFGE, AFLP, *Staphylococcus aureus* subsp. *anaerobius*, Goat, Morel’s disease

## Abstract

*Staphylococcus aureus* subsp. *anaerobius* is the etiological agent of the Morel’s disease in sheep and goats. The disease presents with subcutaneous abscesses, located mainly in the superficial lymph nodes. Forty-one isolates of *S. aureus* subsp. *anaerobius* were collected from two outbreaks of the Morel’s disease in Poland in years 2006–2008. Analysis of DNA *Sma*I digests by PFGE showed that 35 of 41 isolates belonged to the same PFGE type, identical to the type strain of *S. aureus* subsp. *anaerobius* ATCC 35844, confirming high level of clonality of the species. The DNA patterns of the remaining identical 6 isolates, different from the reference strain only by two bands, were found closely related. Genotyping performed with AFLP technique revealed two clonal groups including 16 and 25 isolates, respectively. The study indicated that AFLP technique might be a better discriminatory tool for genetic analysis of *S. aureus* subsp. *anaerobius* isolates, when compared to PFGE.

## Introduction

*Staphylococcus aureus* subsp. *anaerobius* is the etiological agent of Morel’s disease, called also the abscess disease, in sheep and goats. The disease manifests itself with the abscesses located almost exclusively in the superficial lymph nodes or in their close vicinity. The bacterium is closely related to the much more common and medically important pathogen—*S. aureus* subsp*. aureus*. The main distinguishing feature is complete inability of *S. aureus* subsp. *anaerobius* to grow under aerobic conditions and lack of catalase activity, which seems to be associated with the pathogenicity of this microorganism (De la Fuente et al. 2010). Methods of genetic analysis developed for *S. aureus* subsp. *aureus* may also be applied to the epidemiological investigation of genetic relationships between isolates of *S. aureus* subsp. *anaerobius*. To date, two different DNA fingerprinting techniques, namely pulsed-field gel electrophoresis (PFGE) and multilocus sequence typing (MLST), have been utilized. They revealed very high homogeneity of isolates coming from Spain, Italy, Denmark, Sudan and Poland (Elhaj and El Sanousi 2005; De la Fuente et al. 2011; Szaluś-Jordanow et al. 2011).

Amplified fragment length polymorphism (AFLP) is a quite novel three-step DNA fingerprinting, developed in early 1990s (Vos et al. 1995). It has been successfully applied to detecting polymorphisms in DNA of many different human and animal pathogens (Elderle et al. 1999; Kokotovic et al. 1999; Gzyl et al. 2005), including *S. aureus* subsp. *aureus* (Cuteri et al. 2004; Gonano et al. 2009). Moreover, it proved to yield results comparable with PFGE and MLST techniques (Melles et al. 2007). Nevertheless, *S. aureus* subsp. *anaerobius* has never been analyzed with this method.

Therefore, the study was carried out to compare discriminatory potential of PFGE and AFLP techniques, when applied to the analysis of genetic profile of *S. aureus* subsp. *anaerobius*.

## Materials and methods

### Pathogen isolation

All 41 *S. aureus* subsp*. anaerobius* strains were obtained in two goat herds in which outbreaks of Morel’s disease were observed in 2006. Both herds had been monitored for 17 months between 2006 and 2008 (Szaluś-Jordanow et al. 2010). At that time, the first herd was visited 9 times and the second herd 7 times, and swabs were collected from all 44 goats in which mature abscesses were detected with clinical examination. Twenty-eight swabs were collected in the first herd, and 26 of them turned out to harbor *S. aureus* subsp*. anaerobius*. In the second herd, 16 swabs were collected and *S. aureus* subsp*. anaerobius* was isolated in 15 cases. The remaining three isolates appeared to be *Corynebacterium pseudotuberculosis*.

The type strain ATCC 35844 of *S. aureus* subsp. *anaerobius* was obtained from LGC Standards. The swabs were cultured on Columbia agar with 5 % sheep blood. Incubation was conducted for 48 h at 37 °C under aerobic and microaerophilic conditions. After isolation of pure cultures, identification of *S. aureus* subsp. *anaerobius* was initially performed basing on the growth only in microaerophilic conditions, microscopic examination (presence of Gram-positive cocci) and biochemical properties (positive coagulase test, negative catalase and clumping factor tests). Biochemical identification was conducted using API-Staph ID (Biomerieux).

### PFGE

Twenty strains, obtained during the initial phase of the study, have already been analyzed using PFGE, and the results were published elsewhere (Szaluś-Jordanow et al. 2011). The remaining 21 strains were cultured on solid ground with blood and then suspended in saline to obtain the density of 3.5 on McFarland scale, centrifuged and mixed with 150 μl of PIV buffer and 150 μl of liquid agarose. After the solidification, the plugs were placed in EC buffer and incubated at 37 °C for 4 h. Then the plugs were incubated in ESP solution at 50 °C overnight, washed in TE buffer and stored in 1 ml TE buffer at 4 °C. Next, each plug was placed in 100 μl restriction buffer for 15 min. Then each plug was transferred into 250 μl of buffer containing 10 U of *Sma*I and incubated for 3.5 h at 37 °C without shaking. The running parameters of the electrophoresis performed with the CHEF-DR II system electrophoresis cell (Biorad) were as follows: initial pulse—5 s, final pulse—40 s, voltage—6 V/cm, time—20 h and temperature—14 °C. The gels were stained with ethidium bromide for 30 min, then washed in distilled water for 30 min, photographed under UV light and documented in the system VersaDoc (Biorad) (Elhaj and El Sanousi 2005). Dendrogram showing relationship strains under study was drawn by cluster analysis with similarity matrices calculated from the Dice product–moment correlation coefficient and UPGMA algorithm (Everitt 1993).

### AFLP

Preliminary screening of different AFLP protocols based on different enzyme restriction/specific adapter ligation and primer-specific amplification with/without selective bases complementary to nucleotides flanking the restriction sites (Vos et al. 1995; Kokotovic et al. 1999; van Elderle et al. 1999; Gzyl et al. 2005) revealed that most informative AFLP patterns for *S. aureus* subsp*. anaerobius* strains were obtained with *Hin*dIII/*Taq*I comparing to *Apa*I/*Taq*I, *Hin*dIII, *Mfe*I/*Bgl*II, *Pst*I/*Taq*I, *Eco*RI/*Mse*I and *Spe*I/*Eco*RI sets tested. Briefly, in the protocol applied to all strains under study, 200 ng of *S. aureus* subsp*. anaerobius* DNA was digested with the *Hin*dIII (BioLabs Inc.)/*Taq*I (Gibco BRL), heat-treated and ligated with adapters T4DNA ligase (BioLabs Inc.). Restriction fragments tagged with specific adapters were then selectively amplified with primers (van Elderle et al. 1999). Each amplification reaction mixture contained 5 μl of 10 times diluted sample, 0,5 μM of Cy5-labeled primer, 1,7 μM of non-labeled primer, 120 μM of dATP, dGTP, dCTP, dTTP each, 0.3 U of AccuTaq-LA DNA polymerase, 3.0 mM of MgCl_2_, 50 mM of TrisHCl, 15 mM of ammonium sulfate and 0.1 % Tween 20. Amplification was performed in the Biometra thermal cycler in conditions published previously (van Elderle et al. 1999). After completion of the PCR, 5 μl of each reaction tube mixture was mixed with 3 μl of loading dye denatured at 90 °C for 3 min and separated through a ReproGel High Resolution (GE Healthcare Bio-Sciences AB, Uppsala) in 0.5× TBE buffer on an ALFexpress DNA sequencer (AmershamPharmacia Biotech). Separation was done at 1,500 V, 60 mA, 25 W for 420 min at 55 °C. To evaluate intra- and inter-gel differences and identity levels, a fluorescein-labeled molecular size marker and an external reference strain were used as external size markers. Stored fluorograms were analyzed with the GelCompar Software Version 3.1 (Applied Maths, Kortrijk, Belgium). Dendrogram for cluster analysis was based on similarity matrices calculated from the Pearson product–moment correlation coefficient and UPGMA algorithm (Everitt 1993).

## Results

**Fig. 1:**
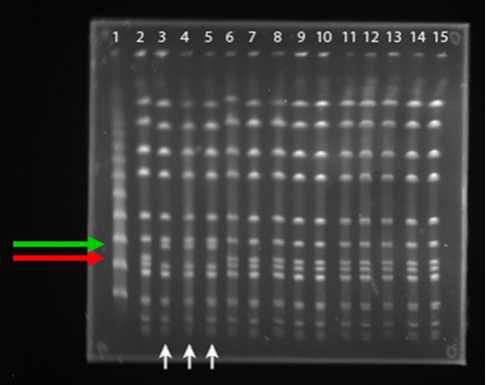
Macrorestriction analysis of chromosomal DNA of *S. aureus* subsp*. anaerobius* isolates by PFGE, *line 1* molecular weight marker, *lines 2*–*14* isolates of *S. aureus* subsp*. anaerobius*, *line 15* reference strain of *S. aureus* subsp*. anaerobius* DSM 20714 (*vertical arrows* indicate the isolates different from the others by two bands, the *upper horizontal arrow* indicates the place of the additional band, and the *bottom horizontal arrow* indicates missing band)

In PFGE 35 of 41 tested isolates proved to be a single clone A, identical to the type strain of *S. aureus* subsp*. anaerobius* ATCC 35844. The remaining 6 isolates (112, 611, 272, 941–5, 101 and 130), designated B, differed in the profile from the reference strain only by two bands and were found closely related (Fig. 1). Genetic similarity for strains under study, which represented two different PFGE profiles as evaluated by cluster analysis, was found 68 %.

**Fig. 2:**
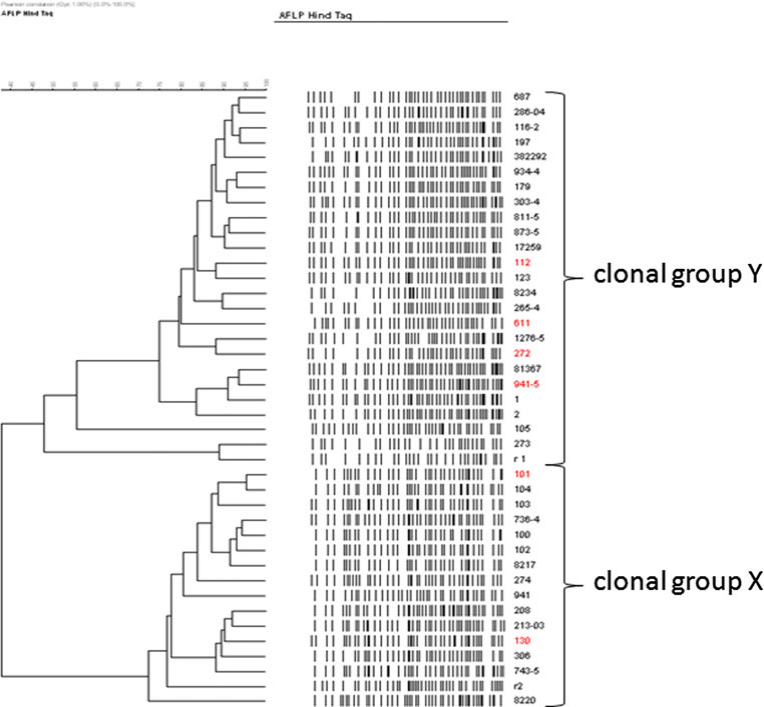
UPGMA dendrogram constructed from AFLP fingerprints showing genetic relationships between *S. aureus* subsp*. anaerobius* isolates. *Red numbers* indicate six different strains in PFGE method (color figure online)

Twenty-three AFLP profiles were identified at the genetic similarity level of 90 %, as overall intra- and inter-gel similarity levels for the external reference strain were 97 and 90 %, respectively. Two clonal AFLP groups, X and Y, could be distinguished, containing 16 and 25 isolates, respectively (Fig. 2). Among X and Y clonal groups identified, 9 and 14 different AFLP patterns were identified, respectively. Genetic similarity of AFLP patterns of isolates belonging to the clonal group X was higher (72 %) than of those belonging to the clonal group Y (47 %). The overall genetic similarity of the isolates was 38 %. In the AFLP clonal group Y and X, 4 and 2 strains belonging to PFGE group B were found, respectively.

## Discussion

The relationship between isolates of *S. aureus* subsp. *anaerobius* was analyzed by PFGE using S*ma*I endonuclease. Previously, PFGE was found to be useful for differentiation of both *S. aureus* subsp*. aureus* (Annemüller et al. 1999) and *S. aureus* subsp*. anaerobius* isolates (Elhaj and El Sanousi 2005; De la Fuente et al. 2011). According to the classification of relationship among isolates by Tenover et al. (1995), 35 of 41 isolates tested in our study were a single clone, identical to the type strain of *S. aureus* subsp*. anaerobius* ATCC 35844.

The remaining six isolates, which differed from the reference strain only by two bands (Fig. 1), were found closely related. Tiny differences detected by PFGE might result from point mutations, for example, substitution, insertion or deletion of single nucleotides, which could possibly occur in the period of time when disease was present in the herd. Our study confirmed high level of clonality of the species reported previously by Elhaj and El Sanousi (2005) and very recently by De la Fuente et al. (2011). While the former study was conducted on only 6 isolates out of which 4 belonged to the same clone, the latter study included as many as 94 isolates, obtained from four different countries, and hence, its results seem to be very reliable.

Higher level of genetic differentiation of analyzed *S. aureus* subsp*. anaerobius* strains in comparison with PFGE was obtained using AFLP technique, as overall genetic similarity of the isolates was 38 %. Two AFLP clonal groups of different genetic similarity levels identified might suggest that two different clones are actually successfully disseminating among goat herds in Poland. Both AFLP and PFGE are considered to yield comparable results in genetic typing of *S. aureus* (Melles et al. 2007); nevertheless, no clear distinction could be made with PFGE. Given that the analysis allowed us to discriminate different DNA profiles at the 90 % identity level, AFLP technique seems to be of higher resolution than PFGE in determining genetic profile of *S. aureus* subsp*. anaerobius*. As in PFGE fingerprinting, strains were differing only by two band positions; both profiles defined among collection under study should be rather considered as subclones. This might be supported by independent position of strains belonging to B PFGE profile within two clusters on the AFLP dendrogram.

Concluding, AFLP technique was found more discriminatory when compared to PFGE fingerprinting. Hence, it may be a useful add-on tool in epidemiological investigation of Morel’s disease.
